# Explainable AI-Driven Analysis of Radiology Reports Using Text and Image Data: Experimental Study

**DOI:** 10.2196/77482

**Published:** 2025-10-14

**Authors:** Muhammad Tayyab Zamir, Safir Ullah Khan, Alexander Gelbukh, Edgardo Manuel Felipe Riverón, Irina Gelbukh

**Affiliations:** 1 Centro de Investigación en Computación (CIC) Instituto Politécnico Nacional (IPN) Ciudad de México, CDMX Mexico; 2 Department of Cell Biology Center for Research and Advanced Studies of the National Polytechnic Institute Ciudad de México, CDMX Mexico

**Keywords:** explainable AI, radiology, natural language processing, SHAP, LIME, artificial intelligence, Shapley Adaptive Explanations, Local Interpretable Model-Agnostic Explanations

## Abstract

**Background:**

Artificial intelligence (AI) is increasingly being integrated into clinical diagnostics; yet, its lack of transparency hinders trust and adoption among health care professionals. The explainable artificial intelligence (XAI) has the potential to improve the interpretability and reliability of AI-based decisions in clinical practice.

**Objective:**

This study evaluates the use of XAI for interpreting radiology reports to improve health care practitioners’ confidence and comprehension of AI-assisted diagnostics.

**Methods:**

This study used the Indiana University chest x-ray dataset containing 3169 textual reports and 6471 images. Textual data were being classified as either normal or abnormal by using a range of machine learning approaches. This includes traditional machine learning models and ensemble methods, deep learning models (long short-term memory network), and advanced transformer-based language models (GPT-2, T5, LLaMA-2, and LLaMA-3.1). For image-based classifications, convolutional neural networks, including DenseNet121 and DenseNet169, were used. Top-performing models were interpreted using XAI methods SHAP (Shapley Adaptive Explanations) and Local Interpretable Model-Agnostic Explanations to support clinical decision making by enhancing transparency and trust in model predictions.

**Results:**

The LLaMA-3.1 model achieved the highest accuracy of 98% in classifying the textual radiology reports. Statistical analysis confirmed the model’s robustness, with Cohen κ (k=0.981) indicating near-perfect agreement beyond chance. Both the chi-square and Fisher exact tests revealed a highly significant association between the actual and predicted labels (*P*<.001). Although the McNemar Test yielding a nonsignificant result (*P*=.25) suggests a balanced class performance, the highest accuracy of 84% was achieved in the analysis of imaging data using the DenseNet169 and DenseNet121 models. To assess explainability, Local Interpretable Model-Agnostic Explanations and SHAP were applied to the best-performing models. These models consistently highlighted that the medical-related terms such as “opacity,” “consolidation,” and “pleural” are clear indications for abnormal findings in textual reports.

**Conclusions:**

The research underscores that explainability is an essential component of any AI systems used in diagnostics and is helpful in the design and implementation of AI in the health care sector. Such an approach improves the accuracy of the diagnosis and builds confidence in health workers, who in the future will use XAI in clinical settings, particularly in the application of AI explainability for medical purposes.

## Introduction

### Background

Artificial intelligence (AI) has the potential to significantly improve diagnostic accuracy, efficiency, and reliability in radiology. Traditional radiology relies heavily on the expertise and subjective judgment of individual radiologists [1], which can be inconsistent and limited when processing large volumes of complex data [2]. AI methods can efficiently analyze vast textual and imaging datasets, but their adoption in clinical practice depends on transparency and interpretability, which are critical for building trust among health care practitioners [3]. This requirement has led to the development of explainable artificial intelligence (XAI), which focuses on both predictive performance and interpretability [4].

Radiology reports include textual narratives describing patient conditions and medical images such as x-rays, CT scans, or MRIs [5]. Most prior studies have analyzed either textual reports or images independently, and multimodal approaches remain limited and insufficiently interpretable. In this study, we analyze text and images separately, applying XAI techniques to each modality. This approach allows clinicians to understand the features driving AI predictions in a modality-specific manner, which is critical for accurate and trustworthy decision-making [6-9].

For textual analysis, natural language processing (NLP) methods extract clinically relevant information from radiology reports [5]. XAI applied to text highlights keywords or phrases influencing AI predictions, helping clinicians verify outputs [10-12].

For image analysis, convolutional neural networks (CNNs) identify critical visual features in radiology images [6-8]. XAI techniques such as Local Interpretable Model-Agnostic Explanations (LIME) or Grad-CAM (Gradient-Weighted Class Activation Mapping) applied to images provide interpretable visual explanations, allowing radiologists to assess the validity of AI predictions [13,14].

Analyzing text and images separately offers several advantages. It allows modality-specific insights, supports clinical education, identifies rare or abnormal findings, and provides independent evaluation of textual and imaging contributions to AI predictions. By making AI outputs interpretable for each modality, clinicians can validate AI suggestions against established diagnostic protocols, improving both confidence and clinical workflow integration [12-14].

In this research, we aim to evaluate the role of XAI models in improving the trustworthiness and interpretability of textual report classification and image analysis. We use NLP techniques by using the up-to-date LLaMA-3.1 language model to classify radiology reports into normal and abnormal categories, achieving 98% accuracy. Statistical validation showed strong model reliability, with a Cohen κ of 0.981 indicating perfect agreement, and both chi-square and Fisher exact test revealing a significant association (*P*<.001). For image analysis, we fine-tuned a DenseNet169 model for image analysis and resulting in an accuracy of 84%. By using these models, we have created a strong approach to interpret radiological information, benefiting from both text and image analysis strengths. In this study, the modalities were analyzed separately to allow clear modality-specific interpretability, which provides a robust foundation for future multimodal frameworks. Such frameworks can integrate textual and imaging features to more closely replicate the complementary reasoning process used by clinicians in practice. To enhance interpretability, we applied SHAP (Shapley Adaptive Explanations) and LIME on the best-performing textual and image models’ outputs. These XAI methods provide insights into how features (words or image regions) influence the model’s decisions, allowing clinical experts to assess whether the AI rationale aligns with clinical reasoning.

The primary objective of this study is to evaluate the role of XAI in enhancing transparency and trust in AI-assisted diagnostics, with a focus on radiology reports and chest x-ray images. Specifically, this study aims to (1) apply the state-of-the-art deep learning as well as transformer models to evaluate both text radiology reports and chest x-ray images separately on the Indiana University dataset, (2) use SHAP and LIME separately on the best performance models with text and image modalities, (3) confirm clinical relevance of the explanations (eg, the existence of such keywords as “opacity” and “consolidation” in text and the identification of pathologic areas on images), and (4) demonstrate explainability to improve clinical trust, interpretability, and the potential acceptance of AI in radiology.

### Related Work

The use of AI in radiology has accelerated over the past few years, with AI being able to assist clinicians in making decisions by automatically interpreting images and analyzing the reports. The traditional radiology workflow relies on the experience and opinion of a radiologist; hence, it can lead to variability in diagnostics and even result in a delay in diagnosis [15].

### Image Analysis in Radiology

Deep learning, particularly CNNs, has shown remarkable performance in detecting abnormalities in medical images. For example, CheXNeXt applied a DenseNet121-based CNN to identify pneumonia in chest x-rays, achieving accuracy comparable to radiologists [16]. Grad-CAM, an XAI technique for image data, generates heatmaps that highlight regions contributing most to model predictions. It has been widely applied in medical imaging tasks, including tuberculosis, breast cancer, and pneumonia detection, providing clinicians with insights into whether models focus on anatomically and diagnostically relevant areas [17].

Despite these advancements, most XAI methods for imaging focus on local explanations rather than providing a global understanding of model behavior. Moreover, few studies integrate image XAI with textual analysis, and clinical validation remains limited.

### Textual Analysis in Radiology

Parallel to image analysis, NLP has advanced the interpretation of radiology reports. Early approaches, such as bag-of-words or TF-IDF (term frequency–inverse document frequency) combined with support vector machines (SVMs) and logistic regression (LR), could not capture contextual relationships [18]. Deep learning models, including recurrent neural networks (RNNs) and bidirectional long short-term memory networks (Bi-LSTMs), improved performance by modeling sequential dependencies in clinical narratives. The advent of transformer-based models, such as Clinical-BERT (Bidirectional Encoder Representations from Transformers) and Bio-BERT, further enhanced text understanding by leveraging pretraining on biomedical corpora [19,20]. The newest large language models (LLMs), including GPT-2, T5, LLaMA-2, and LLaMA-3.1, demonstrate strong generalization across medical domains with minimal fine-tuning, enabling accurate report classification and information extraction [20].

XAI has emerged to address the critical limitation of deep learning models: the lack of interpretability. SHAP assigns feature importance scores using cooperative game theory, while LIME provides local approximations for individual predictions [21,22]. SHAP has been applied to tasks such as sepsis prediction [23], whereas LIME has facilitated clinical validation of NLP models on pathology reports [24]. However, most studies provide explanations for individual predictions only, without offering a global understanding of model behavior.

### Limitations and Research Gaps

Along with these advancements, the given approaches face several issues. The majority of XAI provide explanations for individual predictions but fail to provide a global perspective on model behavior. Most of the research focuses on text or images without combining both XAI methods. Finally, there is not much clinical validation of XAI systems yet because many studies focus on technical evaluation instead of how well they work in the real world and how well health care professionals accept them [25].

The existing studies reveal the gap in the lack of systematic application and validation of multiple XAI methods, both to textual and image-based radiology data, and the use of the same dataset, although SHAP and LIME, their comparative performance and compatibility with clinical knowledge are under discussion in an integrated environment. Besides, the results of new transformer models were released recently (such as LLaMA-3.1), when applied to clinical NLP problems, specifically explainability. Additionally, statistical validation is used to check how good a model is, which helps make sure that AI solutions for health care are reliable.

## Methods

### Study Design

This study followed a structured methodology comprising several steps, beginning with data acquisition and preprocessing, followed by model training for textual and image data separately, and concluding with the XAI technique to interpret the results. This study was conducted in line with the TRIPOD+AI (Transparent Reporting of a multivariable prediction model for Individual Prognosis Or Diagnosis–Artificial Intelligence) guidelines (checklist provided in Multimedia Appendix 1).

### Dataset Description

**Figure 1:**
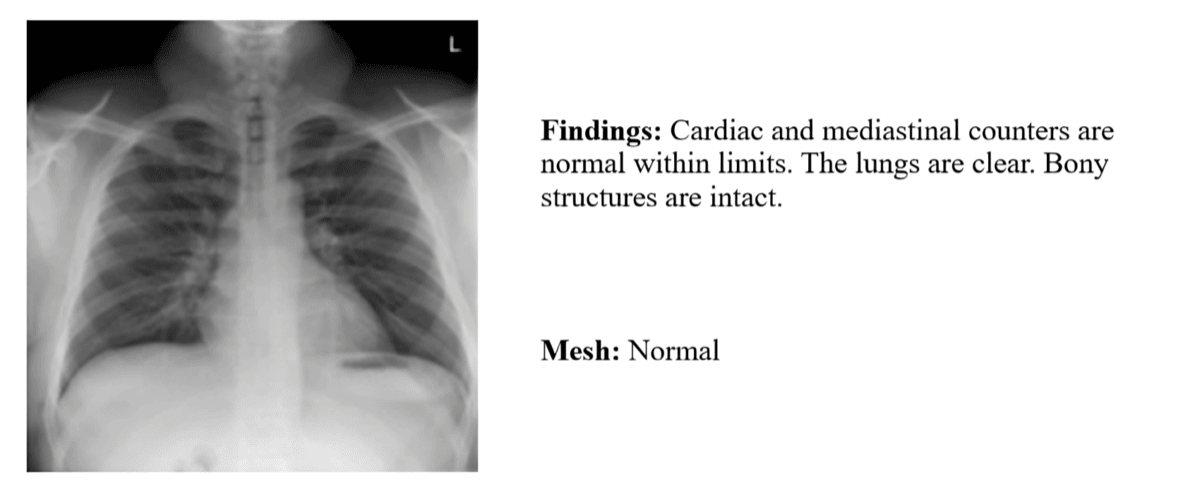
A chest x-ray image and its report with findings and impression.

For this study, the dataset was sourced from the Indiana University chest x-ray collection made available in part by the National Library of Medicine’s Open-I program [26-29]. The dataset contains both text-based clinical radiology reports and their associated imaging reports. The reports have been coded into normal and abnormal with the assistance of the MeSH (Medical Subject Headings) keyword. At the beginning of this work, the internal dataset consisted of 3955 samples of textual radiology reports. After a thorough data preprocessing, 3169 samples were left, with 2535 used for training and 634 for testing. For the image dataset, 7470 images were acquired in the first stage, but after duplicates and deficits in the sample designs were removed, 6471 samples were retained. These were further subdivided into 5177 images for training and 1294 images for testing. All images belong to a set of x-ray reports and are in PNG graphical format. A sample dataset is shown in Figure 1.

### Preprocessing

#### Preprocessing for Textual Reports

Processing of text data included removal of duplicate reports and then their tokenization, where individual sentences were broken down into smaller tokens that are words, with the help of the NLTK library. The next step performed on the text was to make it in lower case, to remove stop words using the NLTK English stop-word list, and lemmatization using WordNetLemmatizer to make sure that the data was preserved and enhanced for NLP tasks.

#### Preprocessing for Image Reports

For image data preprocessing, all the images were adjusted to 255×255 pixels by using bilinear interpolation to ensure standardization across the dataset. The pixel values were normalized in the range 0, 1 (binary) to enhance model efficiency, and repeated diagnostic reports were removed.

#### Textual Reports Classification Models

Several classification methods were used in this task on the textual resource [30], among them it is worth mentioning that SVM classifier is for classes separation, LR is where binary response is examined, naïve bayes is commonly used for classification with probabilities of being in certain class, AdaBoost is for boosting weak classifiers, decision trees is for the processing of decisions, k-nearest neighbor (KNN) is for working with classification by proximity, random forest is when there are large amounts of data, and multilayer perceptron uses neural networks for forecasting. To enhance the performance of machine learning models, we applied ensemble techniques, specifically hard and soft voting classifiers, by aggregating the predictions of multiple machine learning models. In the hard voting approach, each model casts a single vote for the predicted class label, and the final decision is made by majority voting. While soft voting, the classifier outputs class probabilities instead of discrete predictions, and these predictions are averaged, and the class with the highest average probability is selected for the final decision. Four deep learning architectures were put into place, in particular, RNNs aimed at learning inputs sequentially, as well as the Bi-LSTM model based on transformer, incorporating sequential learning and contextual embedding in actual classification of texts [31]. In this study, transformer models were used: BERT, RoBERTa (Robustly Optimized Bidirectional Encoder Representations from Transformers Pretraining Approach), ALBERT-base-v2 (A Lite Bidirectional Encoder Representations from Transformers), and DistilBERT (Distilled Version of Bidirectional Encoder Representations from Transformers) to analyze confidence levels in radiology reports. BERT has been effective in improving the detection of abnormalities by incorporating terminologies and contexts related to the medical field. RoBERTa and ALBERT base version 2 aided in the detection of preabnormalities by pretraining and optimizing power related to computational detection [32]. The availability of DistilBERT further enhanced the efficiency of the clinical decision-making system.

Strengths of various LLMs were also studied, with an emphasis on understanding and rating the degree of abnormalities that could be identified in radiological reports. The models included were XLNet, T5, GPT-2, LLaMA-2, and LLaMA-3.1. The XLNet model improved understanding of language using autoregressive pretraining, along with capturing bidirectional context, while the T5 model treated individual tasks of NLP as problems of converting text into text. GPT-2 was quite rapid in natural language generation, and LLaMA-2 and LLaMA-3.1 further advanced this capacity and trained on high volumes of data, performing language tasks of high complexity. Each model was fine-tuned with tailored hyperparameters as summarized in Table 1 to ensure fairness in evaluation rather than one-size-fits-all approaches.

**Table 1:** Summary of models, definitions, and hyperparameter configuration.

Model	Definition	Key parameters	Purpose of the study
Logistic regression	Linear model estimating probabilities using a logistic function.	Solver=‘liblinear’, C=1.0, max_iter=1000	Baseline classifier for comparison.
Random forest	Ensemble of decision trees using bagging and feature randomness.	n_estimators=200, max_depth=20, criterion=‘gini’	Robust baseline to handle nonlinearities.
Decision tree	Tree-based model splitting data by features iteratively.	max_depth=15, min_samples_split=2	Simple interpretable model for benchmarking.
SVM^a^	Finds a hyperplane maximizing the margin between classes.	kernel=‘rbf’, C=2.0, gamma=‘scale’	Strong baseline for high-dimensional text features.
KNN^b^	Classifies samples by majority vote of nearest neighbors.	n_neighbors=7, metric=‘minkowski’	Benchmark nonparametric learner.
AdaBoost	Boosting weak learners sequentially to minimize errors.	n_estimators=150, learning_rate=0.5	Boosting baseline for imbalanced classes.
MLP^c^	Feedforward neural network with multiple layers.	hidden_layers=(256,128), activation=‘relu’, optimizer=Adam(lr=0.001), epochs=30	Captures nonlinear text or image relations.
Naïve bayes	Probabilistic model assuming feature independence.	MultinomialNB(alpha=1.0)	Classic text classification baseline.
Soft voting	Combines model probabilities for the final prediction.	Weights tuned based on validation accuracy	Ensemble stability.
Hard voting	Combines model class labels by majority voting.	Equal weight	Ensemble diversity.
CNN^d^	Convolutional layers capture spatial or textual patterns.	filters=128, kernel=3, optimizer=Adam(lr=1e-4), dropout=0.5	For image or text local feature extraction.
LSTM^e^	Sequential model capturing long-term dependencies.	hidden_units=128, dropout=0.3, optimizer=Adam(lr=1e-3), epochs=20	Sequential feature modeling in text.
Bi-LSTM^f^	Processes sequences forward and backward for a richer context.	hidden_units=128, dropout=0.3	Improved sequential modeling.
GRU^g^	Simplified LSTM with fewer parameters.	hidden_units=128, dropout=0.3	Efficient sequential modeling.
RNN^h^	Basic recurrent network for sequential data.	hidden_units=64, optimizer=Adam(lr=1e-3)	Baseline sequential learning.
BERT^i^	Transformer model pretrained on bidirectional context.	batch_size=16, lr=2e-5, epochs=3	Contextual embeddings for text.
RoBERTa^j^	Robustly optimized BERT variant.	lr=2e-5, batch_size=16, epochs=3	Better optimization for text classification.
DistilBERT^k^	Lightweight distilled version of BERT.	lr=3e-5, batch_size=32, epochs=4	Efficient transformer baseline.
ALBERT^l^	Parameter-sharing optimized BERT variant.	lr=2e-5, epochs=3	Memory-efficient transformer.
XLNet	Autoregressive transformer capturing bidirectional context.	lr=2e-5, batch_size=8, epochs=3	Strong baseline for sequential tasks.
GPT-2	Transformer-based generative language model.	lr=1e-5, epochs=3, adapter-tuning	Used for generative evaluation of reports.
T5	Text-to-text transformer for multiple NLP^m^ tasks.	lr=3e-5, epochs=3, batch_size=16	For sequence-to-sequence modeling.
LLaMA-2	Lightweight LLM^n^ optimized for research tasks.	LoRA/QLoRA fine-tuning, rank=8, α=16, lr=2e-4	Efficient fine-tuning on radiology text.
LLaMA-3.1	Latest LLaMA with enhanced reasoning.	QLoRA fine-tuning, rank=8, 4-bit quantization, lr=2e-4	Advanced LLM for domain text analysis.

^a^SVM: support vector machine.

^b^KNN: k-nearest neighbor.

^c^MLP: multilayer perceptron.

^d^CNN: convolutional neural network.

^e^LSTM: long short-term memory network.

^f^Bi-LSTM: bidirectional long short-term memory network.

^g^GRU: gated recurrent unit.

^h^RNN: recurrent neural network.

^i^BERT: Bidirectional Encoder Representations from Transformers.

^j^RoBERTa: Robustly Optimized Bidirectional Encoder Representations from Transformers Pretraining Approach.

^k^DistilBERT: Distilled Version of Bidirectional Encoder Representations from Transformers.

^l^ALBERT: A Lite Bidirectional Encoder Representations from Transformers.

^m^NLP: natural language processing.

^n^LLM: large language model.

#### Images Reports Classification Models

Three traditional classification algorithms were used on the analyzed image data; SVM was used for optimal class discrimination, and LR was applied for class probabilities estimation, with random forest assisting in 50,000 structures, limiting the over-fitting problem [26]. For deep learning, several models were fine-tuned [27], including ResNet50, ResNet152, EfficientNet B7, VGG16, DenseNet121, DenseNet169, and used their architecture for sharper feature extraction. In order to enhance the interpretability of model outputs, techniques of XAI LIME and SHAP were used [28] on the best model results for textual and image reports. With the help of such models, the reasons behind the AI classification were made clear to researchers and medical professionals, so that AI decisions would not contradict their clinical findings. These explanations were generated from input text and model output probabilities, helping to determine which specific terms triggered normal and abnormal findings. For image data, we use LIME on the pixel-level input features to generate heat maps highlighting the most significant region influencing the DenseNet169 model decisions. This visualization allowed us to verify whether the model focuses on clinically relevant areas of chest x-rays. It was shown that by incorporating image analysis in addition to text analysis, a powerful method was developed that takes the advantages of both modalities. Models developed within the unified framework significantly outperformed their individual counterparts in terms of diagnosis accuracy and interpretability on a diverse dataset of radiology reports. Such an approach demonstrates how XAI can bring the operational aspects of sophisticated AI models into a clinic and improve diagnosis and patient outcomes. Table 2 details the optimization strategies and hyperparameter configurations for image classification data to ensure fair and robust performance comparisons.

**Table 2:** Fine-tuning and hyperparameter settings for image models.

Model	Definition	Key hyperparameters
Logistic regression	Linear classifier predicting probabilities from weighted features.	C=0.1-10, Solver=lbfgs, Max iter=100
SVM^a^	Finds optimal margin separating classes in feature space.	C=0.1 Kernel=linear, Gamma=scale
Random forest	An ensemble of decision trees aggregating predictions.	Trees=100, Max depth=50, Min split=2
CNN^b^	Learns hierarchical spatial patterns from images.	Input=255×255, Batch=32, LR=2e-5, Epochs=10
ResNet50/152	Deep residual network with skip connections.	Pretrained, LR=2e-5, Batch=16, Epochs=3, Fine-tune last 3 layers
EfficientNet B7	Scales network depth, width, and resolution efficiently.	Pretrained, LR=2e-5, Batch=16, Epochs=30
VGG16	Sequential convolutional network with uniform architecture.	Pretrained, LR=2e-5, Batch=32, Epochs=30, Dropout=0.5
DenseNet121/169	Densely connected layers promote feature reuse.	Pretrained, LR=2e-5, Batch=16, Epochs=30, Dropout=0.5, Fine-tune last dense block

^a^SVM: support vector machine.

^b^CNN: convolutional neural network.

#### XAI Methods

XAI methods SHAP and LIME were applied to textual and image reports for the best-performing models to ensure transparency. This study did not yet implement a joint multimodal model; instead, each modality was analyzed independently to provide a foundation for future integration.

#### About LIME

To explain individual predictions, LIME uses an interpretable model that estimates the model locally.

LIME gives weight to particular words that have an impact on categorization for text. For images, it emphasizes the super-pixels that have a high impact on the prediction.

#### About SHAP

SHAP uses Shapley values of cooperative game theory to explain how each feature affects the outcome of any model. In the case of text classification, for example, normal and abnormal radiology reports, SHAP can be used to display how individual words can contribute to the estimated class. Positive values of SHAP increase the probability of the predicted class, whereas negative values move the prediction toward the alternative one. In the case of image-based models, SHAP can analyze the importance of regions of the image by assessing the variation in the probability of prediction when a region is masked.

#### Evaluation Metrics

The performance evaluation metrics for the classifiers for this work are evaluated on the basis of precision, recall, *F*_1_-score, and accuracy.

Accuracy is the proportion of all correct predictions (true positives and true negatives) out of the total predictions.

Accuracy=(TP + TN)/ (TP + TN + FP + FN)

Where TP are the number of true positive classes, TN are the number of true negative classes, FP are the number of false positive classes, and FN are the number of false negative classes.

Recall is defined as the proportion of true positive predictions out of all actual positives.

Recall=TP/ (TP + FN)

Precision is the proportion of true positive predictions out of all positive predictions made.

Precision=TP/ (TP + FP)

*F*_1_-score is the harmonic mean of precision and recall, balancing the two.

### Statistical Tests

Different statistical tests were performed on the confusion matrices for best best-performing models for images and text data to validate the significant performance of the models.

Cohen κ test is used to measure the interrater agreement between the model predictions and ground truth, which can be computed as:

Kappa=(observed agreement − expected agreement) / (1 − expected agreement).

To calculate the significant relationship between actual labels and predicted labels, we used the chi-square test.

Chi-square=sum of [(observed − expected)^2^/ expected].

The Fisher exact test is an alternative to the chi-square test. It is used when the samples are smaller in size, and it calculates the probability of being as extreme as you can by considering the null hypothesis as true.

The McNemar test measures the performance of 2 models on the same dataset with the formula:

(|b − c| − 1)^2^ / (b + c), where b and c are counts for disagreements.

To statistically validate the performance of the best-performing models, multiple tests were applied to their confusion matrices. Cohen κ was used to measure agreement between the model predictions and the ground truth, accounting for agreement that could occur by chance, which is particularly important in datasets with class imbalance. Chi-square and Fisher exact tests assessed whether there was a significant association between predicted and actual labels, with Fisher Exact applied in cases of small sample sizes to ensure validity when some confusion matrix cells had low counts. The McNemar test was used to compare paired model predictions on the same dataset, focusing on the counts of disagreements to determine whether one model significantly outperformed another. These tests served as complementary validation to accuracy and *F*_1_-scores, confirming consistency and ruling out random agreement. Their interpretive value is supportive rather than providing new insights.

### Ethical Considerations

This study did not involve direct interaction with human participants. We used the public dataset Indiana University Chest x-ray dataset, which consisted of radiographic images and associated reports. The dataset documentation states that all of the data was completely anonymized before being made public, and additionally does not require any more ethical approval. Consequently, informed consent, compensation, and institutional review board approval did not apply to this study.

## Results

Results for textual data and image data were calculated separately by applying different models.

### Models Results for Textual Reports

The analysis of text-based radiology machine learning classifiers and ensemble methods revealed significant performance differences across the classifiers. In this research, we evaluate the various machine learning, deep learning, transformers, and LLMs for textual radiology reports performance as shown in Table 3. The performance evaluation metrics for the classifiers for this work were evaluated based on precision, recall, *F*_1_-score, and accuracy.

**Table 3:** Classification algorithms’ performance metrics for textual data.

Models	Precision (%)			Recall (%)			*F*_1_-score (%)			Accuracy (%)
	Macro	Weighted	Macro		Weighted	Macro		Weighted		
Logistic regression	95	94	88		94	91		94	94	
Random forest	93	93	85		92	88		92	92	
Decision tree	83	88	83		88	83		88	88	
SVM^a^	94	94	89		94	91		94	94	
KNN^b^	75	86	83		81	77		82	81	
AdaBoost	89	91	83		91	86		90	91	
MLP^c^	91	93	90		93	91		93	93	
Naïve bayes	93	92	85		92	88		92	92	
Soft voting	96	95	91		95	93		94	95	
Hard voting	96	95	89		95	92		94	95	
CNN^d^	93	95	92		95	93		94	92	
LSTM^e^	95	95	91		95	94		95	95	
Bi-LSTM^f^	96	96	92		96	96		96	96	
GRU^g^	94	95	91		95	92		96	95	
RNN^h^	92	94	92		94	92		94	94	
BERT^i^	92	95	94		95	93		95	95	
RoBERTa^j^	96	96	96		96	96		96	96	
DistilBERT^k^	93	96	94		95	93		95	95	
ALBERT^l^-base	96	96	96		96	96		96	96	
XLNET	94	96	95		96	95		96	96	
GPT-2	93	96	94		96	94		96	96	
T5	95	96	95		97	96		97	97	
LLaMA-2	98	98	98		98	98		98	98	
LLaMA-3.1	98	99	98		99	98		99	98	

^a^SVM: support vector machine.

^b^KNN: k-nearest neighbor.

^c^MLP: multilayer perceptron.

^d^CNN: convolutional neural network.

^e^LSTM: long short-term memory network.

^f^Bi-LSTM: bidirectional long short-term memory network.

^g^GRU: gated recurrent unit.

^h^RNN: recurrent neural network.

^i^BERT: Bidirectional Encoder Representations from Transformers.

^j^RoBERTa: Robustly Optimized Bidirectional Encoder Representations from Transformers Pretraining Approach.

^k^DistilBERT: Distilled Version of Bidirectional Encoder Representations from Transformers.

^l^ALBERT: A Lite Bidirectional Encoder Representations from Transformers.

Soft voting and hard voting classifiers reported the highest precision of 96% and accuracy of 95%, thus the most reliable approaches within the current study scope. LR and SVM were also quite efficient, attaining scores of 94% for both. However, KNN performed the least with an accuracy of 81%. As a rule, the combined classifier approaches, in comparison to the singular classifiers, were more effective, giving improved precision, recall, and *F*_1_-scores. From the deep learning perspective, the Bi-LSTM model was ranked first among the models with a macro precision of 96%, macro recall of 92%, macro *F*_1_-score of 96%, and an accuracy of 96%. However, among the deep learning algorithms, the RNN model was the least performing. For transformer-based models, RoBERTa and ALBERT-base achieved the best performance measures with macro precision, macro recall, macro *F*_1_-score, and accuracy all at 96%. DistilBERT worked well but not quite as successfully, with a macro precision score of 93% and accuracy of 95%. Table 3 shows that LLaMA-3.1 performed the best with macro-precision, macro-recall, and macro *F*_1_-score of 98%, weighted *F*_1_-score 99% in addition to 98% accuracy. Our results outperform those reported by Wiggins et al [29], demonstrating improved accuracy and overall performance because LLaMA-3 has a larger capacity and optimized transformer architecture, which capture complex patterns and long-range dependencies. Its pretraining on a massive, diverse corpus, combined with domain-specific fine-tuning and careful hyperparameter optimization, enhances understanding of clinical terminology and report structure. Additionally, LLaMA-3 efficiently handles longer sequences, resulting in higher accuracy and stronger agreement with ground truth.

The LLaMA-2 followed with an accuracy of 98%, macro *F*_1_-score of 98%, and weighted *F*_1_-score 98%. T5 came next with 97% accuracy, while the performance of both XLNET and GPT-2 was on par with 96% accuracy. The above results suggest that the new approach goes a notch higher in performance than previous studies, making remarkable improvements in the accuracy and overall performance of the system.

### Models’ Results for Image Reports

To classify the image data for radiology reports, we applied machine learning and deep learning algorithms, and the results are shown in Table 4.

**Table 4:** Classification algorithms’ performance metrics for radiology image data.

Models	Precision (%)			Recall (%)			*F*_1_-score (%)			Accuracy (%)
	Macro	Weighted	Macro		Weighted	Macro		Weighted		
Logistic regression	66	67	65		66	65		66	66	
SVM^a^	63	65	64		65	64		65	65	
Random forest	55	59	54		59	56		59	59	
CNN^b^	60	64	62		64	63		64	64	
ResNet50	61	63	61		64	62		64	64	
ResNet152	71	72	70		72	71		72	72	
EfficientNet B7	61	64	61		62	63		64	64	
VGG16	80	81	80		80	80		81	81	
DenseNet121	81	84	82		83	82		84	84	
DenseNet169	81	84	82		83	82		84	84	

^a^SVM: support vector machine.

^b^CNN: convolutional neural network.

The machine learning classifiers for image data showed in Table 2 that LR outperformed SVM and random forest models; LR with an accuracy of 66%, macro precision of 66%, macro recall of 65%, and macro *F*_1_-score of 65%. Among the deep learning classifiers, EfficientNet B7 achieved a notable accuracy of 72%, macro precision of 71%, macro recall of 70%, and macro *F*_1_-score of 71% and obtained more results than ResNet50 and ResNet152. The highest performance was seen with DenseNet169 and DenseNet121, which reached an accuracy of 84%, macro precision of 81%, macro recall of 82%, and macro *F*_1_-score of 82%, highlighting their superior capability in handling complex image data. In our study, the performance benchmarks for image analysis have been significantly improved compared to previous research, which reported an accuracy of 79.16% [27]. We fine-tuned the DenseNet169 on our specific radiology image dataset, allowing the model to adapt its feature representations to the unique characteristics of the data. Key hyperparameters, such as learning rate, batch size, number of frozen layers, and optimizer settings, were systematically tuned. We used some of the same models as the prior study but incorporated additional techniques such as random crops, flips, and color adjustments to enhance model generalization. These techniques have led to a substantial improvement in our results.

### Statistical Analysis for Textural Reports

**Figure 2:**
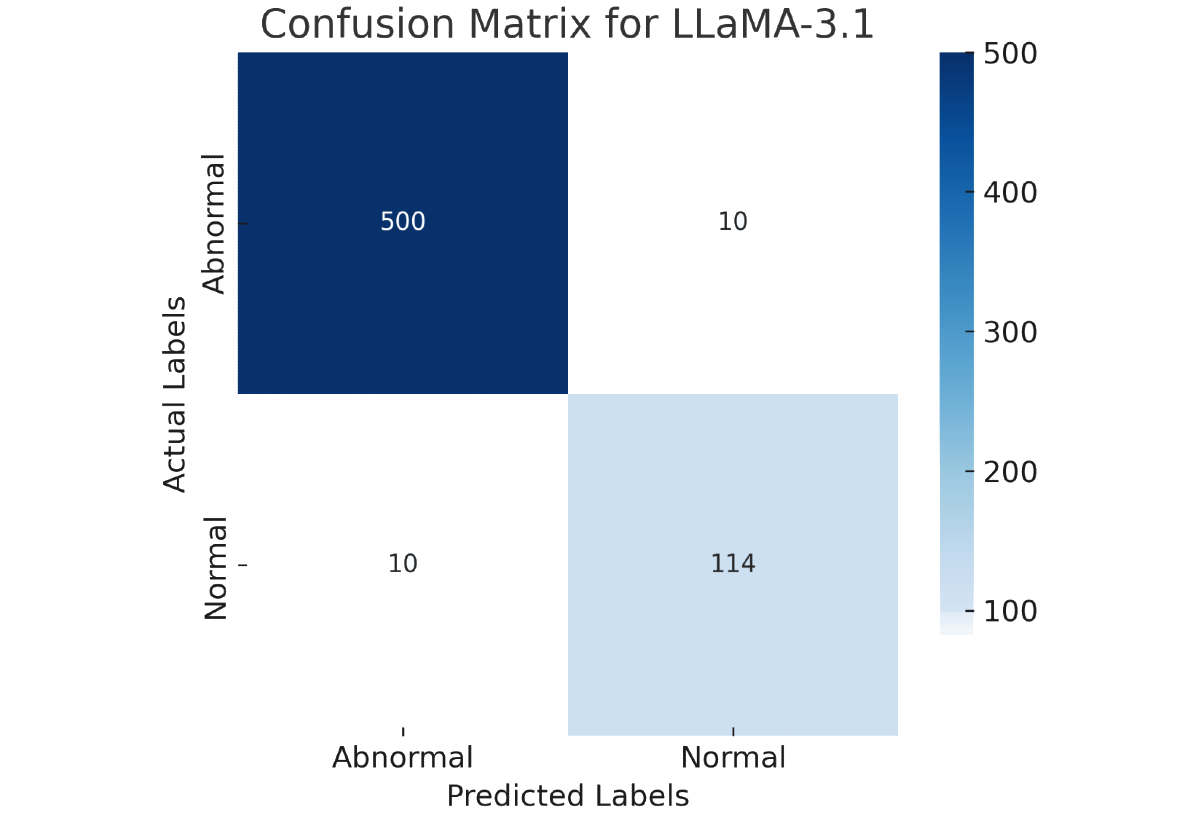
Shown is the best confusion matrix for the best-performing model LLaMA-3.1 for textural radiology reports.

The classification performance of the top model LLaMA-3.1 was evaluated using a confusion matrix, as shown in Figure 2. The confusion matrix offers a comprehensive perspective of the model’s predictions, particularly its ability to classify 2 target classes, normal and abnormal. Of 634 cases, 500 were classified as normal (true positive) and 114 as abnormal (true negative); only 20 samples were classified as missed. The overall metrics for models are calculated as shown in Figure 2.

To evaluate whether the model errors are symmetrically distributed between false positives and false negatives, different statistical tests were applied as shown in Table 5, which include the McNemar test, chi-square test, Cohen κ test, and Fisher exact test. These tests provide complementary validation of the confusion matrix results. Cohen κ confirmed strong agreement beyond chance, while chi-square and Fisher exact indicated significant associations between predictions and ground truth. The McNemar test showed balanced errors between classes. While these results are consistent with the accuracy and *F*_1_-scores, they do not provide independent evidence of clinical utility but rather confirm the robustness of the model’s performance on this dataset.

**Table 5:** Statistical significance tests for top top-performing model LLaMA-3.1.

Test	Formula	Value	*P* value	Interpretation
McNemar test	χ² = ((|b − c| − 1)²) / (b + c)	—^a^	.62	0.25
Chi-square test	χ² = Σ((O – E)² / E)	Very high (≫1000)	<.001	Strong dependence between predicted and actual classes
Cohen κ test	κ = (Po – Pe) / (1 – Pe)	κ = 0.981	—	Perfect agreement beyond chance
Fisher exact test	Based on hypergeometric probability	—	<.001	Significant association between actual and predicted

^a^Not available.

Table 5 provides a set of statistical tests used to evaluate the performance of the LLaMA-3.1 model in classifying normal and abnormal instances. The McNemar test, with a *P* value of .25, shows that there may be no statistically huge difference among the types of classification errors made by the model. The chi-square test yielded very high test statistics with a *P* value less than .001, strongly indicating a significant association between actual and predicted labels. In other words, the model predictions are highly aligned with ground truth labels.

Additionally, the Cohen κ score from Table 3, 0.981, demonstrated near-perfect agreement between the model prediction and actual labels. This reflects a very high level of consistency and reliability in the model classification performance. Finally, Fisher exact test, which is used in full for small sample sizes, also yielded a significant *P* value (<.001), further confirming the model’s predictive performance is statistically significant. Overall, all 4 tests collectively suggest that LLaMA-3.1 is a highly accurate and dependable model for classifying normal and abnormal case reports.

### Error Analysis for Image Reports

**Figure 3:**
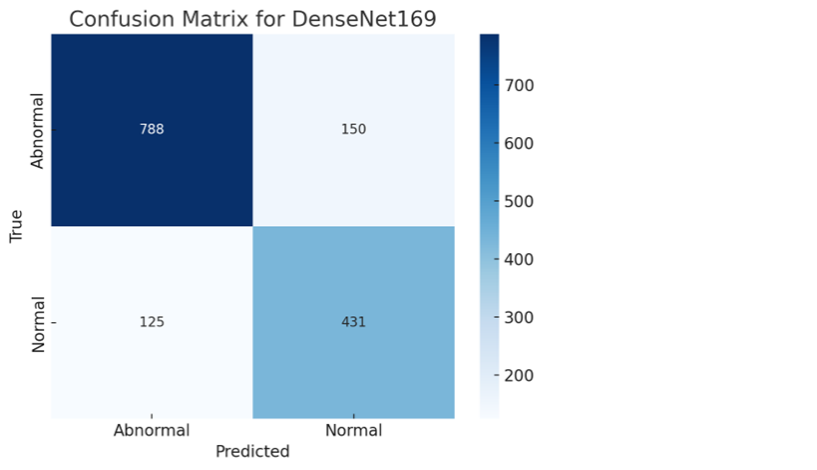
Shown is the best confusion matrix for the best-performing model, DenseNet169, for images of radiology reports.

As illustrated in Figure 3, the DensetNet169 confusion matrix shows that the model correctly classifies 788 abnormal and 431 normal cases. However, it misclassifies 125 normal cases as abnormal (false positives) and 150 abnormal cases as normal (false negatives).

These results highlight the model’s effectiveness in identifying abnormal instances with high precision.

### Results for XAI

To enhance the interpretability of our AI models used for classifying both textual radiology reports and image data, we used XAI techniques LIME and SHAP on the best results models for textual and image test data. These techniques provide detailed justifications of the parts or regions that influenced the model’s decisions, making the AI’s classifications more transparent and easier to understand for researchers and medical experts**.**

**Figure 4:**
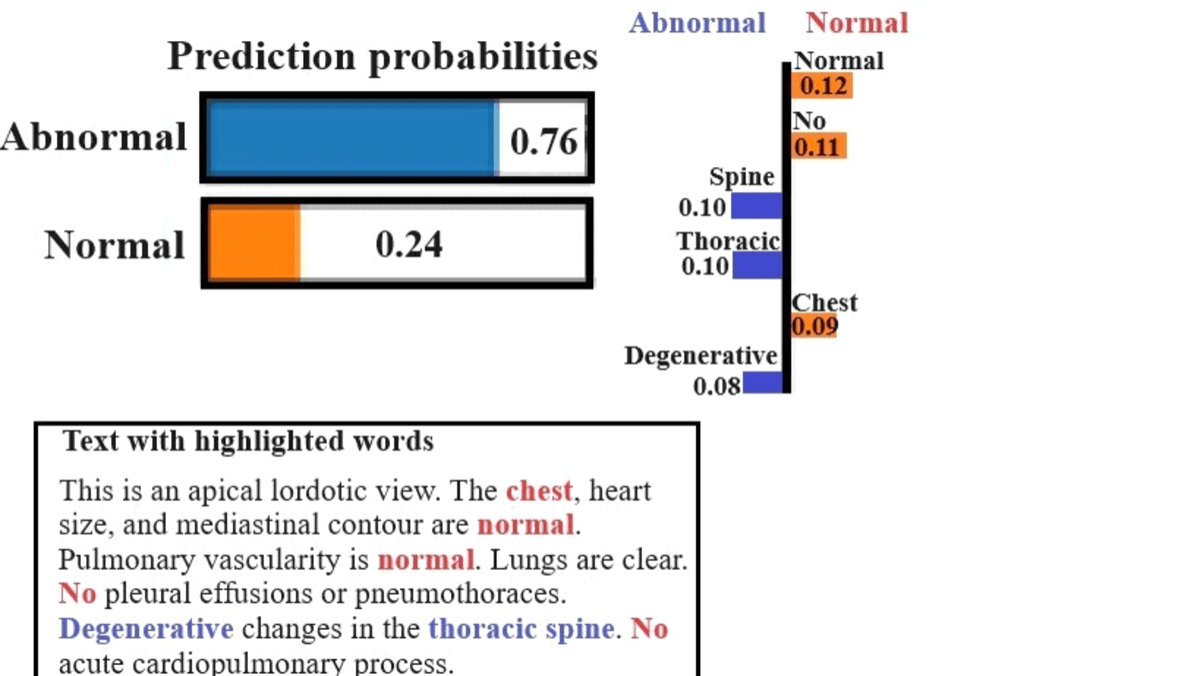
LIME results for abnormal radiology report. LIME: Local Interpretable Model-Agnostic Explanations.

In Figure 4, an interpretation analysis of LIME for a radiology sample report is given. The LIME model places a high probability of 0.76 that the report would be abnormal and 0.24 as to whether it would be normal. The result of this classification is critical in the clinic, where a wrong diagnosis may result in inappropriate treatment or a failure to diagnose a health issue. In blue, words such as spine, thoracic, chest, and degenerative have been included as the most influential features toward the abnormal prediction of the model. These terms are usually associated with structural or pathological observations, which substantiate the choice of the model and agree with the common radiological signs of abnormal results. Conversely, words such as “normal” and “no,” in orange color, are related to the normal class. These words can indicate the lack of findings, which justifies the lower probability assigned to the normal class. The highlighted text at the bottom of the figure further refines how these words are distributed throughout the radiology report, offering a transparent view of how a model intercepts the context. This is essential for clinical explainability, allowing radiologists to verify whether the model predictions are grounded in medically relevant features.

**Figure 5:**
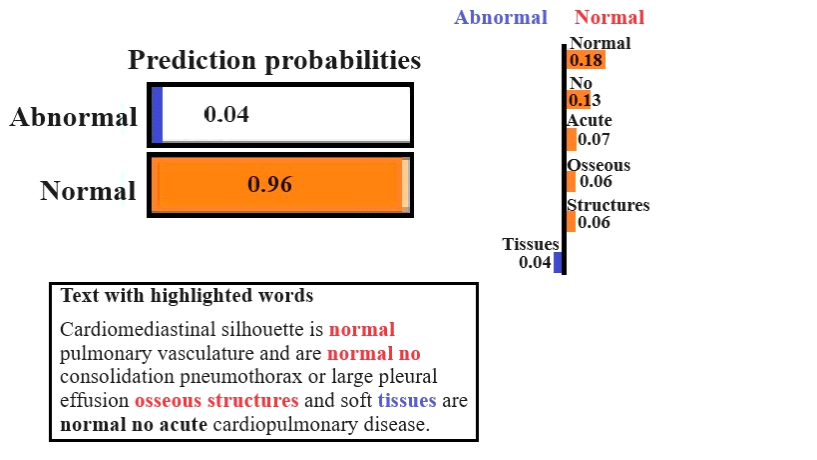
LIME results for a normal radiology report. LIME: Local Interpretable Model-Agnostic Explanations.

Figure 5 illustrates a radiology report interpretation where LIME assigns a high probability of 0.96 to the normal class, with only 0.04 probability for the abnormal class. This strongly indicates the model’s confidence in classifying this report as a normal radiology finding. Words such as “normal,” “no,” and “acute” highlighted in orange significantly contributed to the normal classification. These terms repeatedly appeared in the report and reflect the absence of any abnormal findings. These are the typical language cues that radiologists use to confirm the normal case, and the model successfully aligns its prediction with them. Terms such as “tissues” are highlighted in blue, suggesting a minor contribution to the abnormal findings. The lower part of Figure 5 provides a visualization of how these words are distributed across the textual report. This helps interpret the reasoning behind the model decision, showing the model appropriate weight on reassuring normal findings while discounting potentially ambiguous terms. This level of interpretability is crucial in the clinical domain, as it allows health care professionals to audit the model’s reasoning and ensure alignment with medical judgments. Figure 5 not only confirms the model accuracy but also validates its decision-making process in handling routine, noncritical radiology reports.

**Figure 6:**
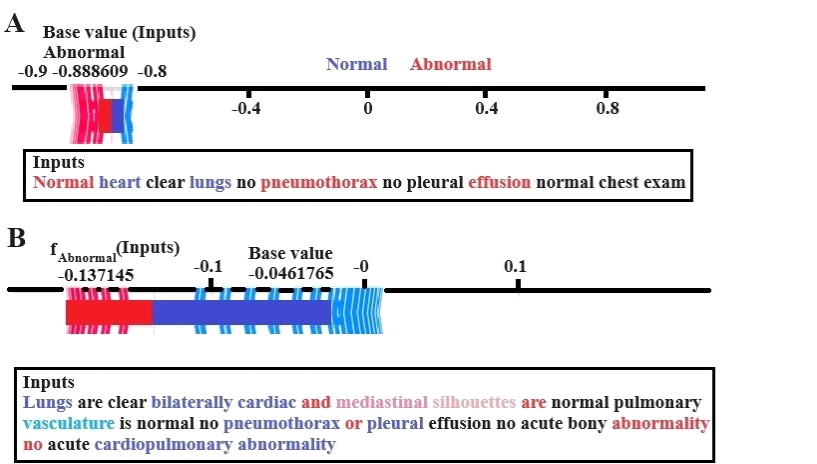
SHAP results for (A) normal and (B) abnormal radiology reports. SHAP: Shapley Adaptive Explanations.

Figure 6 illustrates the contribution of different input features to the predictions of a model regarding normal versus abnormal medical reports. These plots serve as a powerful, interpretable tool, offering a fine-grained look at the model reasoning process for specific radiology reports. In Figure 6A, the SHAP plot shows that overall prediction is skewed toward the abnormal finding, with the base value of –0.888 indicating the model’s confidence in this outcome. Keywords such as “pneumothorax” and “effusion” are highlighted in red, which indicates positively toward the abnormal classification. Meanwhile, the terms “lungs” and “heart” appear in blue, opposing the abnormal prediction. Despite the presence of some reassuring words, the presence of abnormality-related terms dominates, driving the model’s final decision, and also the base value of prediction starts at around –0.9, indicating a strong likelihood of an abnormal report. In Figure 6B, the base value starts near 0, showing a more balanced prediction. SHAP base value starts at around –0.046, and the prediction is very close to 0. Features such as “lungs,” “bilaterally cardiac,” “vasculature,” and “cardiopulmonary abnormality” also push the prediction for the normal, which are highlighted in blue. In contrast, terms such as “bony” and “abnormality” contribute toward the label abnormal, but not strongly enough to influence the outcome significantly.

**Figure 7:**
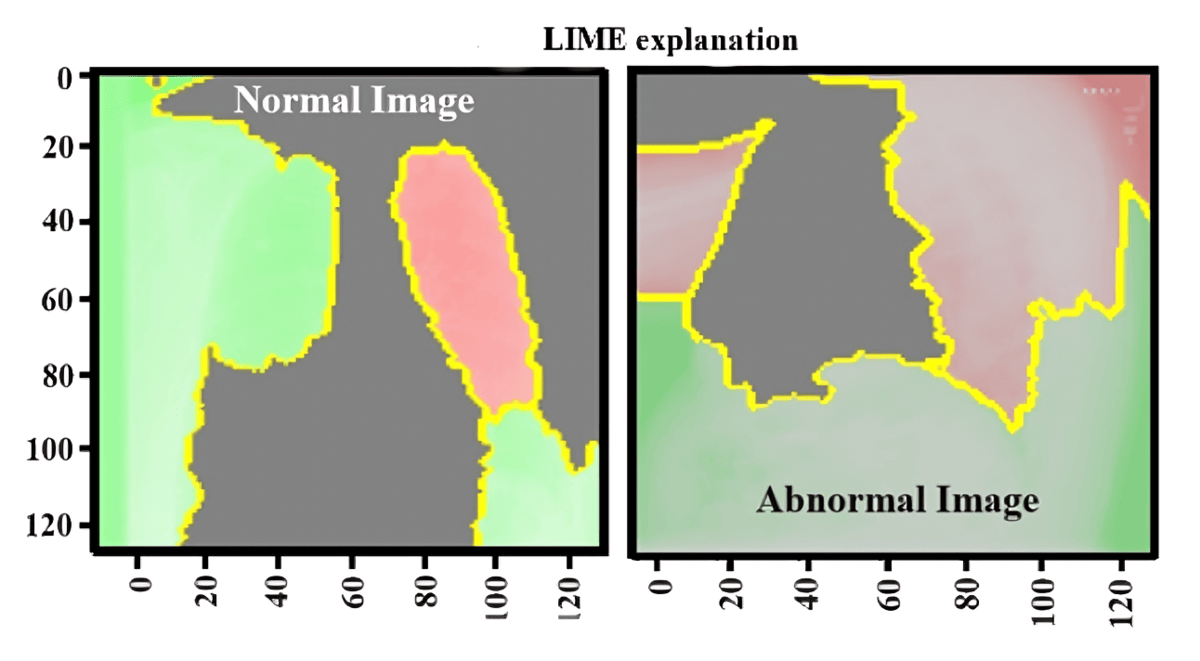
LIME explanation for the normal and abnormal image reports dataset. LIME: Local Interpretable Model-Agnostic Explanations.

Figure 7 presents the LIME analysis, visualizing regions in medical images contributing to the prediction of normal and abnormal reports. The left side displays a normal image, where red and green areas represent parts of the image that the model considered significant for identifying a normal image. The red area may seem important, but the model correctly identifies its normal image. On the right-hand side, the abnormal image highlights regions contributing to the abnormal prediction, with more red areas indicating potential issues. The yellow outlines show the border of these influential areas. LIME visualizes these explanations by segmenting the image and showing how much impact the model decision has, providing insight into why the model made its prediction for normal and abnormal findings.

### Enhancing Clinical Utility With XAI

SHAP and LIME were used to interpret model outputs; they serve other purposes, too. In clinical practice, radiologists often face ambiguous cases where features are overlapping, such as early signs of pneumonia and mild pulmonary edema. With SHAP, global and local feature importance gives radiologists the ability to view text or images as phrases, “increased opacity,” and “no acute findings” that triggered the AI’s decision. This indicates whether the model is leveraging medically applicable regions as opposed to irrelevant, misleading ones. SHAP, for example, helped confirm that model bias due to excess reliance on template phrases was not classified too heavily using them. LIME, on the other hand, gave out local explanations that were more intuitive by showing the words or pixels that were decisive for the model’s decision, aiding in false positive or negative detection. The information allows radiologists to trust or contest AI’s outputs, reducing uncertainty in diagnostics and increasing confidence in informed decisions, especially in nuanced situations where clinical discretion is pivotal.

### Complementary Roles of SHAP and LIME

To enhance the transparency of model prediction, we used each SHAP and LIME, which serve complementary roles in explaining AI version behavior, while each methods’ intention was to interpret complicated machine learning outputs.

SHAP is based on the game theory idea and provides both local and global explanations by calculating the contribution of each feature to the final prediction using Shapley values. It guarantees consistency and a clear mathematical basis, which makes it useful for knowing how a model behaves throughout the whole dataset and identifying the maximum influential functions as usual. LIME, in comparison, specializes in local interpretability by constructing an interpretable linear model around a single prediction to explain the results. It is especially intuitive and beneficial for explaining individual instances, specifically misclassifications or borderline selections, showing which functions contributed at maximum to that prediction.

Using both models allowed us to provide a comprehensive explanation of the model’s decision-making process. SHAP summarized feature importance and overall model reliability, while LIME provided explanations for individual clinical predictions in a human-readable format. This dual approach enhances clarity and transparency, particularly for readers or clinicians who may not be familiar with the inner workings of the algorithm. Moreover, while recognizing prevailing trends, we acknowledge their validity in the context of real-world diagnostic applications.

## Discussion

### Principal Findings

This study benchmarked multiple machine learning, deep learning, and transformer models for radiology report and chest x-ray classification, with interpretable models. For textual radiology data, ensemble methods, particularly soft and hard voting classifiers [33], outperformed individual machine learning algorithms [34]. Clinically, this suggests that combining multiple models can produce more reliable predictions, reducing the likelihood of errors from a single classifier [35]. These results are consistent with previous research demonstrating the benefits of ensemble strategies for improving the robustness and performance of recognition systems [36].

For deep learning models, Bi-LSTM [37] efficiently captures sequential dependencies in radiology reports, allowing better contextual understanding of clinical narratives. Transformer-based models such as RoBERTa and ALBERT-base [38] also showed improved performance with domain-specific fine-tuning [39], highlighting that incorporating radiology-specific knowledge enhances both accuracy and interpretability. These models could help clinicians by efficiently summarizing complex report content and identifying subtle patterns, thereby reducing cognitive load and potential human error.

Among the evaluated models, LLaMA-3.1 showed the strongest overall performance. Its value lies not only in accuracy but in producing clinically relevant and interpretable predictions when paired with SHAP and LIME [40,41]. These models have the potential to assist in routine diagnostic tasks where high precision and interpretability are crucial, increasing clinicians’ trust in AI-assisted decision-making. This emphasizes the need to adapt AI tools for specialized medical applications, ensuring their safety and effectiveness in clinical practice [42]. The future of AI in radiology also promises faster disease recognition, more personalized treatment recommendations, and improved patient outcomes [43].

Regarding imaging data, while traditional machine learning classifiers such as SVM [44] performed reasonably well, they could not match the performance of specialized CNN architectures such as EfficientNet B7 [45] and DenseNet169 [46]. DenseNet169 demonstrates the advantage of deep learning in extracting hierarchical and nuanced features from complex medical images, improving diagnostic accuracy.

SHAP and LIME [47] generated explanations that highlighted plausible clinical terms and regions. However, their relevance to practicing radiologists remains untested. Clinical trust cannot be assumed, and structured evaluations with radiologists are required to confirm whether these explanations enhance diagnostic confidence, efficiency, and decision-making.

Although our results demonstrate strong technical performance and interpretable output, their impact on real-world radiology workflows remains untested. In practice, clinical adoption depends not only on accuracy but also on whether AI explanations can integrate seamlessly into existing reporting systems, save time, or improve decision-making under time constraints. These aspects were beyond the scope of this study but are critical for clinical translation.

### Limitations

We acknowledge several limitations in this work. First, the Indiana dataset used for training and evaluation is relatively small, dated, and lacks diversity in terms of patient demographics, imaging modalities, and reporting styles. These characteristics may bias both predictive performance and explainability outcomes. For example, the textual reports often follow templated or simplified structures, which could artificially boost model accuracy while reducing robustness to more varied reporting styles. Similarly, the imaging data may not reflect the diversity of acquisition protocols or disease prevalence seen in modern radiology practice, limiting the transferability of both predictions and explanations. Consequently, while the dataset serves as a useful benchmark, its constraints reduce the applicability of our findings to contemporary, real-world clinical environments. Furthermore, the analysis relied entirely on a single dataset without cross-institutional or external validation, which limits the generalizability of the findings. Another important limitation is that text and images were analyzed separately rather than through a multimodal framework, which does not fully reflect the integrated nature of real diagnostic workflows. Additionally, although SHAP and LIME produced interpretable outputs, their clinical utility remains uncertain because they were not systematically validated with radiologists. In this study, we assumed that highlighted terms and image regions aligned with clinical reasoning, but we did not test whether clinicians found these explanations trustworthy, intuitive, or useful in practice. Without structured user validation, the degree to which XAI outputs improve diagnostic confidence or decision-making remains speculative. The inclusion of a large number of models provided broad benchmarking but lacked a sharply defined hypothesis, weakening the methodological focus. Another limitation is that our explainability analysis relied solely on SHAP and LIME. While these methods are widely used and model-agnostic, they primarily generate local explanations and are known to have limitations such as instability in feature attributions or sensitivity to parameter choices. The absence of complementary global or clinically validated approaches, such as Grad-CAM for imaging data or counterfactual explanations for text, may restrict the interpretive depth and clinical relevance of our findings. Finally, the statistical tests applied (Cohen κ, chi-square, Fisher exact, and McNemar) primarily confirmed results already reflected in accuracy and *F*_1_-score metrics. While useful for robustness checks, they add limited new interpretive value, and their role in demonstrating clinical reliability should not be overstated.

### Future Work

Future research will focus on addressing these limitations to strengthen the clinical value of XAI in radiology. Specifically, we aim to develop multimodal models that integrate textual reports with imaging data to better capture complementary diagnostic information and reflect real-world workflows. We will extend validation across external datasets from different institutions and explore approaches such as domain adaptation and federated learning to enhance cross-site robustness.

To confirm the practical value of AI explanations, we will conduct structured evaluations with radiologists through reader studies, workflow simulations, and integration into reporting systems. These studies will test whether XAI outputs reduce diagnostic uncertainty, improve decision-making compared to existing practices, and fit within the time constraints of clinical workflows. Additionally, usability testing will ensure that explanations are not only technically interpretable but also practical, efficient, and supportive of radiologists’ routine tasks. As a part of future work, we will apply Grad-CAM to image models to generate global visual explanations that align with radiological regions of interest, integrated gradients to provide robust attribute scores, and counterfactual explanation techniques for textual reports to illustrate how minimal changes in phrasing alter predictions. We will test our models on larger and more diverse datasets such as MIMIC-CXR and CheXpert, which include broader patient demographics, varied imaging modalities, and more contemporary reporting styles. This will allow us to assess not only predictive accuracy but also the stability and clinical plausibility of SHAP and LIME explanations across heterogeneous clinical contexts.

### Take Home Points

The key points are as follows:

- These types of techniques, especially modern deep learning and LLMs, increase the effectiveness of diagnosis in the case of textual and image-based radiological data.
- Using XAI for decision support using LIME and SHAP leads to a better understanding of the AI models and their outcome, which implies better clinical decisions.
- The work cites studies that have shown advanced performance improvements over previous work with large models such as LLaMA-3.1 that achieved high accuracy rates, demonstrating a strong appreciation for the use of fine-tuned models in the clinical domain.
- Some weaknesses of this study included the concerns of dataset representativeness, because of challenges of explaining modeling outcomes in real-world clinical practice with respect to the existing methods of XAI approaches, and future directions and improvement of the model are recommended.
- This study emphasizes that there is a need for such integration whereby the intention of those algorithms is about prediction rather than replacing the clinical judgment of the clinicians.

### Conclusions

In conclusion, our study reveals that among machine learning classifiers for textual radiological data, ensemble methods such as soft voting and hard voting achieved the highest precision and accuracy at 96% and 95%, respectively, outperforming individual classifiers such as LR and SVM, which had accuracies of 94%. In contrast, KNN had the lowest performance with an accuracy of 81%. For image data, SVM led among traditional machine learning models with an accuracy of 66%, while DenseNet169 excelled among deep learning classifiers with an accuracy of 84%. Notably, fine-tuned LLMs such as LLaMA-3.1 reached a remarkable accuracy of 98%, with strong agreement (Cohen κ=0.981) and statistical validation (*P*<.001). The McNemar test (*P*=.25) confirmed balance classification, significantly surpassing previous benchmarks in textual data analysis. While these results indicate strong potential for improving diagnostic support, external validation and prospective clinical studies are required to confirm their effectiveness in real-world clinical settings.

## Appendix

Multimedia Appendix 1 TRIPOD+AI checklist.

## Abbreviations

AI: artificial intelligence
BERT: Bidirectional Encoder Representations from Transformers
Bi-LSTM: bidirectional long short-term memory network
CNN: convolutional neural network
DistilBERT: Distilled Version of Bidirectional Encoder Representations from Transformers
Grad-CAM: Gradient-Weighted Class Activation Mapping
KNN: k-nearest neighbor
LIME: Local Interpretable Model-Agnostic Explanations
LLM: large language model
LR: logistic regression
MeSH: Medical Subject Headings
NLP: natural language processing
RNN: recurrent neural network
RoBERTa: Robustly Optimized Bidirectional Encoder Representations from Transformers Pretraining Approach
SHAP: Shapley Adaptive Explanations
SVM: support vector machine
TF-IDF: term frequency–inverse document frequency
TRIPOD+AI: Transparent Reporting of a multivariable prediction model for Individual Prognosis Or Diagnosis–Artificial Intelligence
XAI: explainable artificial intelligence

## Data Availability

The dataset is publicly available through the US National Library of Medicine Open-I platform and can be accessed under a public domain license. The code used in this study is publicly available online [48].
