# Supplementary material for: Explainable AI-Driven Analysis of Radiology Reports Using Text and Image Data: Experimental Study
Source: JMIR Form Res. 2025 Oct 14;9:e77482. doi: 10.2196/77482 (PMC12569488; doi:10.2196/77482)
Supplement: Multimedia Appendix 1 [file formative_v9i1e77482_app1.docx]

| \| **Item** \| \| **Section / Topic** \| **E: Explanation (Requirement)** \| **D: Description (Reported in Study)** \| **Page Number** \| \| --- \| --- \| --- \| --- \| --- \| --- \| \| 1. Title \| \| Title & Abstract \| Identify study as developing/evaluating prediction model, target population, outcome \| Title: *Explainable AI-driven analysis of radiology reports using text and image data: An experimental study* \| 1 \| \| 2. Abstract \| \| Title & Abstract \| Structured abstract per TRIPOD+AI \| Structured with Background, Objective, Methods, Results, Conclusion. Includes dataset, models, and XAI methods (SHAP, LIME). \| 2–3 \| \| 3. Background \| \| Introduction \| Explain background and rationale \| AI in radiology, black-box issues, need for explainability. \| 3–5 \| \| 4. Objectives \| \| Introduction \| State objectives/hypotheses \| Evaluate XAI (LIME, SHAP) on text & image radiology data. \| 5 \| \| 5. Source of Data \| \| Methods – Dataset \| Describe dataset, source \| Indiana University Chest X-ray dataset from Open-I. \| 6 \| \| 6. Participants \| \| Methods – Dataset \| Eligibility criteria \| Reports labeled normal/abnormal using MeSH; duplicates removed. \| 6 \| \| 7. Outcome Definition \| \| Methods \| Define predicted outcomes \| Binary: normal vs abnormal (text and image). \| 7 \| \| 8. Predictors \| \| Methods \| Define predictors/features \| Text features: tokens/embeddings; image features: CNN layers. \| 7 \| \| 9. Sample Size \| \| Methods \| Explain sample size, handling missing data \| Reports: 3,169; Images: 6,471. Split train/test. \| 6–7 \| \| 10. Missing Data \| \| Methods \| Handling missing data \| Removed duplicates/deficient samples; no imputation required. \| 7 \| \| 11. Statistical Analysis \| \| Methods \| Describe metrics, validation \| Accuracy, Precision, Recall, F1; statistical tests (Cohen’s kappa, Chi-square, Fisher’s Exact, McNemar). \| 8-12 \| \| 12. Model Development \| \| Methods \| Detail models/training \| ML (SVM, RF, LR, NB, KNN), DL (CNN, LSTM, BiLSTM), Transformers (BERT, RoBERTa, T5, GPT-2, LLaMA). Image CNNs (ResNet, EfficientNet, DenseNet). \| 7–10 \| \| 13. Model Performance \| \| Results \| Report on model performance \| LLaMA-3.1 98% accuracy; DenseNet169/121 84%. Tables provided. \| 12–18 \| \| 14. Model Explainability \| \| Methods & Results \| Report explainability \| LIME/SHAP applied; examples in Figures 4–7 showing keywords (“opacity”, “consolidation”) and image regions. \| 18–22 \| \| 15. Results – Participants \| \| Results \| Flow of data \| Reports Classification for images and textual reports \| 12-24 \| \| 16. Limitations \| \| Discussion \| Limitations/bias \| Single dataset, imbalance, limited generalizability. \| 24 \| \| 17. Interpretation \| \| Discussion \| Interpret results \| LLaMA-3.1 best for text; DenseNet for images; explainability improved trust. \| 23–24 \| \| 18. Clinical Implications \| \| Discussion \| Relevance to practice \| XAI enhances trust and transparency for clinicians. \| 24 \| \| 19. Conclusion \| \| Conclusion \| Provide conclusion \| Multimodal XAI improves diagnostic support and trust. \| 25-26 \| \| 20. Data & Code \| \| Availability \| Report availability \| Data from Open-I; code on GitHub. \| 26 \| \| 21. Ethics \| \| Ethical Statement \| Ethical approval/consent \| Dataset anonymized, no IRB required. \| 26 \| \| 22. Conflict of Interest \| \| Declarations \| Funding/conflicts \| No conflicts; supported by CONACYT, Microsoft, etc. \| 26 \| \|  \| |
| --- | --- | --- | --- | --- | --- | --- | --- | --- | --- | --- | --- | --- | --- | --- | --- | --- | --- | --- | --- | --- | --- | --- | --- | --- | --- | --- | --- | --- | --- | --- | --- | --- | --- | --- | --- | --- | --- | --- | --- | --- | --- | --- | --- | --- | --- | --- | --- | --- | --- | --- | --- | --- | --- | --- | --- | --- | --- | --- | --- | --- | --- | --- | --- | --- | --- | --- | --- | --- | --- | --- | --- | --- | --- | --- | --- | --- | --- | --- | --- | --- | --- | --- | --- | --- | --- | --- | --- | --- | --- | --- | --- | --- | --- | --- | --- | --- | --- | --- | --- | --- | --- | --- | --- | --- | --- | --- | --- | --- | --- | --- | --- | --- | --- | --- | --- | --- | --- | --- | --- | --- | --- | --- | --- | --- | --- | --- | --- | --- | --- | --- | --- | --- | --- | --- | --- | --- | --- | --- | --- |
